# Reiter's Syndrome

**Published:** 1951-07

**Authors:** A. E. W. McLachlan

**Affiliations:** Director, Venereal Disease Clinics, Bristol


### REITER'S SYNDROME

BY

A. E. W. McLACHLAN, M.B., D.P.H., F.R.S.E.
*Director, Venereal Disease Clinics, Bristol*

Reiter's syndrome comprises:

1. *An abacterial urethral discharge*, frequently associated with dilatation of the renal pelves, less commonly pyelitis, haematuria, prostatitis;

2. *Ocular symptoms*: commonly bilateral conjunctivitis, less frequently episcleritis, iritis or herpes.

3. *Polyarthritis:* and

4. *Muco-cutaneous manifestations:*—hyperkeratosis, balanitis circinata, and circular areas of desquamation on the dorsum of the tongue. There is frequently associated an irregular low-grade pyrexia.

### Aetiology

The aetiology is still in doubt. Reiter in his original case isolated a spirochaete, which he termed S. forans, from the blood stream, and named the disease spirochaetosis arthritica. Subsequent workers have failed to confirm this finding. A diversity of organisms, staphylococci, enterococci, diphtheroids, B. coli, etc., have been isolated by various observers from the urethral discharge. But when meticulous precautions are taken to cleanse the meatus before obtaining the specimens no organisms are found in smears or cultures. These organisms must therefore be regarded as contaminants. Pleuropneumonia-like organisms have been suggested as the possible cause by Smith (1942) and Dienes and Smith (1942) who isolated " L " organisms from the urethral discharge in Reiter's disease, while in 1948 Dienes *et al.* demonstrated this organism in the synovial fluid of two cases, a finding confirmed by Warthin in another patient. Harkness (1944) suggested the possibility of a virus—as evidenced by the finding of inclusion bodies in scrapings from the urethra, conjunctiva, and skin lesions: but later Harkness and Henderson-Begg (1948) isolated " L " organisms in 17 per cent. of forty-one cases of Reiter's disease; at the same time Giemsa-stained smears showed markedly pleomorphic inclusion bodies in the epithelial cells. These cytoplasmic inclusions are compatible with either a virus or a pleuropneumonic infection. Dunham *et al.* (1947) cultivated by egg-allantoic inoculation a filterable agent from the urethral and conjunctival secretion of a case of Reiter's disease. This culture proved pathogenic for mice, 60 per cent. of those inoculated intraperitoneally developing conjunctivitis. Allergy has been suggested by Thiers and Joly (1948) on the basis of an exacerbation of symptoms and fever following the intradermal injections of a small amount of the urethral discharge. While the aetiology still remains uncertain, the presence of " L " organisms in a high percentage of cases of Reiter's disease, and their disappearance following successful treatment suggest that this is the probable causal agent.

### Transmission of Infection and Sexual Incidence

The term Reiter's disease is used indifferently to describe the urethritis-arthritis-conjunctivitis syndrome complicating acute, or following upon apparently cured, dysentery; as well as the essentially similar condition following sexual exposure and abacterial urethritis. There is an extensive literature on both the dysenteric and sexual forms, and apart from the urethritis, which may be little marked in the former group of cases, the systemic sequelae follow the same pattern. It is reasonable to assume therefore that the causal agent can obtain entry into the body by two separate portals, the ulcerated large intestine or the inflamed urethra.

The disease is more prevalent in males. In the "dysenteric" group Paronen (1948) recorded thirty-four infections in females in a total of 344 cases. In the "urethral" group Harkness (1945) could only find nine cases recorded in the literature. The author has only recognized four undoubted cases in females in a series of ninety-five. It is possible that many further cases would be recognized if a search for pleuropneumonic organisms and cytological inclusions were carried out in cases of nongonococcal urethritis, cervicitis, etc., associated with a history of transient conjunctivitis, arthritis, etc.

All the cases recorded in childhood have been in association with dysentery. Corner (1950) reporting one case could only find three other cases under twelve years of age, reported in detail in the literature.

### Clinical Course and Symptomatology

The author's experience has been confined to the sexual group of infections. The frequency of involvement of the various systems was:

| Sex | Urethritis, Prostatitis, cervicitis, etc. | Conjunctivitis or other ocular signs | Arthralgia, Arthritis, etc. | Keratoderma, etc. |
|---|---|---|---|---|
| Male 91 | 91 (100) | 60 (66) | 71 (78) | 28 (31) |
| Female 4 | 4 (100) | 4 (100) | 3 (75) | 1 (25) |

*Percentages in parentheses.*

The clinical course may be extremely variable: there are undoubtedly many mild forms, self-limiting or responding to minimal treatment. Approximately 25 per cent. follow a more severe course, lasting from seven to eight weeks up to three to six months. Less frequently there is a series of major or minor relapses: in these cases, examination of the wife (or consort) has shown nothing beyond a mild cervicitis. That reinfection was a possible explanation of the relapses is supported by freedom from recurrence after treatment of the spouse.

*Urinary Tract Manifestations.* The first symptom usually noted is urethritis which becomes apparent after a long incubation period of from ten to thirty days. In the majority of cases the urethritis is mild, a clear, tenacious urethral discharge being associated with only scanty mucoid urinary threads. There is no accompanying dysuria or increased frequency of micturition and the prostate and vesicles are frequently normal, although 25 per cent. of patients show a symptomless prostatitis. In the remainder of cases there is considerable variation up to the acute type, characterized by marked dysuria, increased diurnal and nocturnal frequency of urination, and a profuse mucopurulent or purulent urethral discharge, occasionally sanious. There is a marked urinary haze; the prostate and seminal vesicles show marked involvement; there is frequently a generalized cystitis; asymptomatic dilatation of the renal pelves is common in this group, while pyelitis, hydronephrosis, and prostatic abscess have been described. There seems to be little relation between the severity of the genito-urinary symptoms and the subsequent systemic sequelae.

*Ocular manifestations.* Conjunctivitis, commonly bilateral, is usually the first sign of generalization, and occurs from one to thirty days after the urethral discharge has been noted. It may be transient, mild and persistent, or severe. Episcleritis is a frequent accompaniment while iritis is found in some 5 per cent. of cases. In cases following a relapsing course, there are frequently recurrences of conjunctivitis or iritis. Conjunctival and corneal herpes have been described.

*Joint Manifestation.* Arthralgia, which frequently precedes the onset of a defined arthritis, may be noted at the time of occurrence of urethral discharge. The pain varies greatly in intensity, is frequently migratory, and may pass off spontaneously, or may become fixed on the development of arthritis. The joints commonly implicated are the same as in arthritis, namely, in descending frequency, the knee, the ankle, the smaller joints of the feet and hands, the elbows, etc. No joint is immune.

In over 80 per cent. of cases the arthritis is acute and polyarthritic and characterized by a rapid effusion into the joint, tense synovial thickening, and reddening and glazing of the overlying skin. Pain is intense. Spontaneous retrogression of the arthritis occurs in from four to six weeks, but this improvement is frequently accompanied by involvement of further groups of joints. Subacute arthritis occurs in approximately 20 per cent. of cases: it may also follow an acute phase or be associated with recurrent attacks of the disease. Pain and effusion are less, but synovial and periarticular thickening gives rise to spindle-shaped swellings. These are well seen in the metacarpo-phalangeal and inter-phalangeal joints. In the later stages, X-ray examination often reveals areas of osteoporosis towards the articular ends of the involved bones. Periosteal reaction is more rare.

*Mucocutaneous manifestations.* Balanitis circinata is possibly the most frequent of the mucocutaneous manifestations, is more common in the uncircumcised, and affects the coronal sulcus and the corona glandis. The appearance varies according to the stage at which it is seen. At first there is a heaping up of layers of scales, in circular areas of from 2 to 10 mm. in diameter: later those are shed, leaving smooth, weeping, circular patches on which fresh scales are laid down. Coalescence of the lesions gives rise to linear or serpigenous configurations. Typical hard keratodermic nodules also occur. On the dorsum of the tongue similar patches of desquamation up to 12 mm. in diameter are found.

Hyperkeratotic manifestations (keratoderma blenorrhagica) are most common on the soles of the feet and on the glans penis, but may occur anywhere on the body. The sequence is first a vesicular eruption which passes into a pustular stage: this is followed by progressive keratinisation in the walls of the pustule. The core becomes dried up, waxy, and cornified. The resulting lesion is raised, limpet-shell-like, hard, and has been aptly compared to "mountains on a relief map". The hyperkeratotic nodules may remain discrete or coalesce to form plaques of varying extent. Soft parakeratotic lesions may also occur, especially on the scalp, consisting of many layers of scales, often raised in the centre, and without cornification or keratinisation. Balanitis circinata is essentially a soft parakeratosis, and the tongue manifestations also fall into this category.

*Pyrexia.* Some degree of fever is found in 80 to 90 per cent. of cases during the period of systemic involvement. The temperature is irregular and swinging up to 100 to 103 deg. F., and is associated with marked cachexia and wasting of the muscles controlling the involved joints. The erythrocyte sedimentation rate is raised in proportion to the severity of the disease. Despite the fever and cachexia, the appetite is unimpaired.

### DIAGNOSIS

Clinically, the complete syndrome presents no difficulties. In incomplete syndromes, how many of the metastatic sequelae are required to complete the diagnosis? Keratoderma alone is sufficient, otherwise the combination of urethritis, ocular, and joint manifestations or balanitis is essential. In many minor cases careful questioning will elicit the transient occurrence of arthralgia or conjunctivitis, while careful examination will reveal residual joint or dermal lesions. Nevertheless, many cases of non-specific urethritis, in which it is impossible at first to make a diagnosis, ultimately prove to be Reiter's disease.

Clinically the disease may be confused with acute rheumatism, metastatic arthritis in association with blood-borne infection, true gonococcal arthritis, etc., while the occurrence of keratoderma may suggest arthropathic psoriasis. The character of the joint fluid, straw coloured, turbid with a cellular content of polymorphonuclear leucocytes and lymhpocytes, and bacteriologically sterile, is of help; while the demonstration of pleuropneumonic organisms or inclusion bodies in the various secretions is confirmatory.

### TREATMENT

Local measures, urethro-vesical irrigation with 1:10,000 solution of oxycyanide of mercury, prostato-vesicular massage, etc., aid in the eradication of the genito-urinary focus. Parenteral administration of calcium salts results in temporary symptomatic relief of the conjunctivitis, and appears to limit the extent of joint effusion. Streptomycin, 4·0 g. daily for seven days is successful in a number of cases: chloromycetin, in a dosage of 0·5 g. two-hourly for six doses, then four-hourly for the next three to four days, has proved even more effective in a short series of cases. Aureomycin is reported to show equally promising results. A number of the chronic relapsing cases react well to gold salts, *e.g.* myocrysin in a dosage of 0·2 g. in five weeks.

The most uniformly successful method of treatment is pyrexial, preferably carried out in a fever cabinet, the temperature being raised to 105 to 106 deg. F., and maintained for from five to eight hours. One to three exposures are adequate: relief of arthritis is spectacular, and is paralleled by the improvement in the eye symptoms. The progress of keratoderma is cut short. The alternative to cabinet-hyperpyrexia is the induction of a series of fevers by the intravenous injection of T.A.B., the commencing dose being 25 million organisms. A temperature of 102 to 104 deg. F. is reached and persists for from two to five hours. Six to twelve fevers may be required, at two to three day intervals. Physiotherapy is required to aid restoration of full joint movement, and recovery of the wasted muscles.

Male, M.8426, *aet.* twenty-five, married.

1943–44 Non-specific urethritis—treated in the Navy with sulphonamides and irrigations—outcome satisfactory.

1949

January Non-specific urethritis, slight urinary haze, no prostato-vesiculitis or other complications.

March Hyperkeratotic balano-posthitis, in coronal sulcus. Condition clear April, 1949—treated sulphathiazole and irrigations.

July Recurrence of non-specific urethritis—clear in one month.

December Slight recurrence of discharge—clear in one month.

In the various examinations, the condition was very similar—scanty mucoid to mucopurulent urethral discharge—slight urinary haze or threads—no prostato-vesiculitis. W.R. and Kahn consistently negative. Smears, pus ±, no gonococci: cultures, no gonococci, no predominant organism.

1950

30th August Recurrence: slight mucopurulent urethral discharge—urine clear—very few threads—prostate and vesicles normal.

3rd September Arthralgia R. knee, conjunctivitis and photophobia.

6th September Acute hydrarthrosis R. knee—synovial thickening.

Over a period of the next six or eight weeks the following joints were involved in sequence, the picture being the same in each case, namely hydrarthrosis with marked synovial thickening, L. knee, R. and L. ankles, R. wrist, L. wrist, carpal and interphalangeal joints of R. hand and then L. hand.

18th September Balanitis circinata.

25th October — Keratodermic lesions, soles of feet, under toe nails, and distal third of leg.

X-ray: joints showed no bone changes.

The erythrocyte sedimentation rate was greatly increased.

Throughout the infection there was a slight, irregular, low-grade fever.

*Treatment*

7.9.50—13.9.50 — *Streptomycin* 4·0 g. daily (total 24·0 g.). Ineffective.

29.9.50—5.10.50 — Distaquaine 0·3 mega unit daily (total 2·1 mega units). Ineffective.

4.10.50—6.11.50 — Intravenous T.A.B. fevers, dosage 12·5 to 100 million organisms.

Gradual improvement, paralleled by return of erythrocyte sedimentation rate to normal. The residual fusiform thickenings of interphalangeal joints resolved completely under short-wave therapy. Now completely fit and is doing full-time work as a general labourer.

### REFERENCES

1. Corner, B. D. (1950). *Arch. Dis. Child.*, *25*, 398.
2. Dienes, L., Ropes, M. W., Smith, W. E., Madoff, S., and Bauer, W. (1948). *New Engl. J. Med.*, *238*, 509, 563.
3. Dienes, L., and Smith, W. E. (1942). *Proc. Soc. Exp. Biol.*., N.Y., *50*, 99.
4. Dunham, J., Rook, J., and Belt, E. (1947). *J. Urol.*, *58*, 212.
5. Harkness, A. H. (1945). *Brit. J. Ven. Dis.*, *20*, 93.
6. Harkness, A. H., and Henderson-Begg, A. (1948). *Brit. J. Ven. Dis.*, *24*, 50.
7. Paronen, I. (1948). *Acta Med. Scand.*, suppl., 212.
8. Reiter, H. (1916). *Dtsch. Med. Wschr.*, *42*, 1535.
9. Smith, W. E. (1942). *J. Bact.*, *43*, 83.
10. Thiers, H., and Joly, L. (1948). *Munch. Med. Wschr.*, *68*, 769.

